# Parkinson's Disease Skin Fibroblasts Display Signature Alterations in Growth, Redox Homeostasis, Mitochondrial Function, and Autophagy

**DOI:** 10.3389/fnins.2017.00737

**Published:** 2018-01-12

**Authors:** Joji M. Y. Teves, Vedanshi Bhargava, Konner R. Kirwan, Mandi J. Corenblum, Rebecca Justiniano, Georg T. Wondrak, Annadurai Anandhan, Andrew J. Flores, David A. Schipper, Zain Khalpey, James E. Sligh, Clara Curiel-Lewandrowski, Scott J. Sherman, Lalitha Madhavan

**Affiliations:** ^1^Graduate Interdisciplinary Program in Applied Biosciences, University of Arizona, Tucson, AZ, United States; ^2^Neuroscience and Cognitive Science Undergraduate Program, Undergraduate Biology Research Program, University of Arizona, Tucson, AZ, United States; ^3^Department of Neurology, University of Arizona, Tucson, AZ, United States; ^4^Pharmacology and Toxicology, University of Arizona, Tucson, AZ, United States; ^5^Graduate Interdisciplinary Program in Physiological Sciences, University of Arizona, Tucson, AZ, United States; ^6^Department of Surgery, University of Arizona, Tucson, AZ, United States; ^7^Department of Medicine, University of Arizona, Tucson, AZ, United States; ^8^The Evelyn F McKnight Brain Institute, University of Arizona, Tucson, AZ, United States

**Keywords:** Parkinson's disease, sporadic, human dermal fibroblasts, oxidative stress, autophagy, mitochondrial function, UVA irradiation

## Abstract

The discovery of biomarkers for Parkinson's disease (PD) is challenging due to the heterogeneous nature of this disorder, and a poor correlation between the underlying pathology and the clinically expressed phenotype. An ideal biomarker would inform on PD-relevant pathological changes via an easily assayed biological characteristic, which reliably tracks clinical symptoms. Human dermal (skin) fibroblasts are accessible peripheral cells that constitute a patient-specific system, which potentially recapitulates the PD chronological and epigenetic aging history. Here, we compared primary skin fibroblasts obtained from individuals diagnosed with late-onset sporadic PD, and healthy age-matched controls. These fibroblasts were studied from fundamental viewpoints of growth and morphology, as well as redox, mitochondrial, and autophagic function. It was observed that fibroblasts from PD subjects had higher growth rates, and appeared distinctly different in terms of morphology and spatial organization in culture, compared to control cells. It was also found that the PD fibroblasts exhibited significantly compromised mitochondrial structure and function when assessed via morphological and oxidative phosphorylation assays. Additionally, a striking increase in baseline macroautophagy levels was seen in cells from PD subjects. Exposure of the skin fibroblasts to physiologically relevant stress, specifically ultraviolet irradiation (UVA), further exaggerated the autophagic dysfunction in the PD cells. Moreover, the PD fibroblasts accumulated higher levels of reactive oxygen species (ROS) coupled with lower cell viability upon UVA treatment. In essence, these studies highlight primary skin fibroblasts as a patient-relevant model that captures fundamental PD molecular mechanisms, and supports their potential utility to develop diagnostic and prognostic biomarkers for the disease.

**Graphical Abstract:**
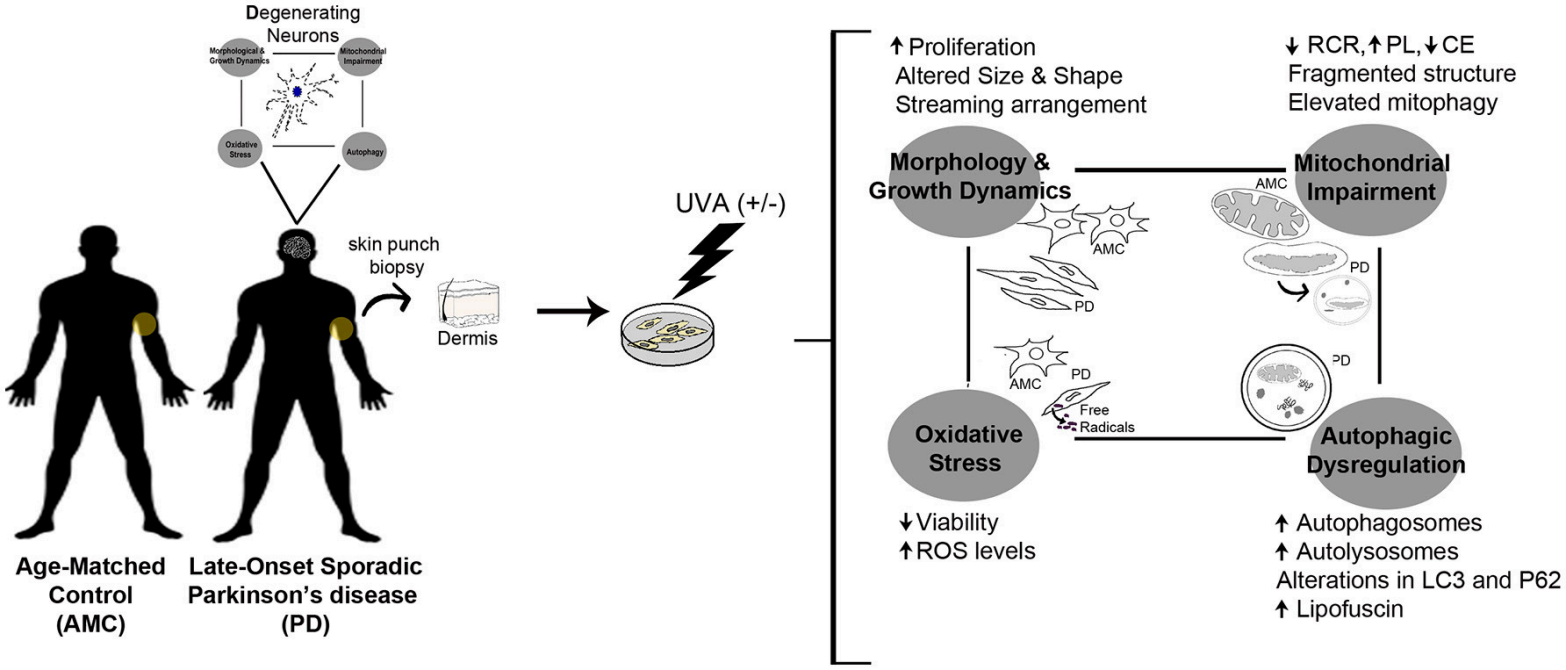
PD skin fibroblasts phenocopy changes typical to degenerating neurons.

## Introduction

Parkinson's disease (PD) is a chronic age-related neurodegenerative disorder, which affects more than 6.5 million people worldwide (Beitz, 2014; Poewe et al., 2017). Although a small proportion of PD has clear genetic linkages, more than 90% of cases are deemed sporadic (or “idiopathic”)—meaning of unknown cause. A major issue faced by PD patients is that although symptomatic therapies are available, there are no current treatments that can slow or halt the progression of the disease (Savitt et al., 2006; Dexter and Jenner, 2013). An important factor impeding therapeutic progress has been the inadequate understanding of PD etiopathogenesis, and its relatively late diagnosis which currently occurs in the clinic at an advanced stage when 60–70% of afflicted neurons have already degenerated (or have become dysfunctional; Kordower et al., 2013). Hence, there is a critical need for human models, which will allow for a robust investigation of how PD develops and support the discovery of reliable biomarkers for early diagnosis of the disease.

It has been proposed that PD has a complex multifactorial etiology, involving many genetic and environmental factors, over the course of aging (Malkus et al., 2009; Gao and Hong, 2011; Cannon and Greenamyre, 2013; Beitz, 2014; Feng et al., 2015; Kannarkat et al., 2015). However, the precise nature of these gene-environment interactions is not well understood, and constitutes an area of high scientific interest. Studies indicate that such complex interactions may ultimately lead to a compromise in fundamental processes that maintain cellular homeostasis, such as mitochondrial function, redox balance, and protein quality control (Malkus et al., 2009; Schapira and Jenner, 2011). In particular, it is understood that interconnected molecular pathways causing mitochondrial dysfunction and impaired bioenergetics, oxidative stress due to excessive production of reactive oxygen species (ROS), and impaired protein degradation, especially through the ubiquitin-proteasome and autophagy-lysosome pathways, can act as common denominators of neuronal death in PD. Thus, a robust PD model and diagnostic can be envisioned as one that would allow for easy monitoring of these fundamental features of the disease.

In this context, some studies have utilized human skin fibroblasts to investigate PD molecular mechanisms (Auburger et al., 2012). Fibroblasts offer an advantage in that they are accessible cells, which can be derived from PD-affected populations to provide a patient-specific culture system to study the disease. In particular, as a primary cell type, fibroblasts retain the specific environmental and aging history, and polygenic risk factors of the patient. Furthermore, although non-neuronal, fibroblasts make dynamic cell-to-cell contacts, similar to neurons, in culture.

Thus far, studies have focused predominantly on analyzing fibroblasts from genetic PD patients, and largely in the context of specific molecular processes of interest (Grünewald et al., 2010; Ambrosi et al., 2014; Mcneill et al., 2014; Norris et al., 2015; Zanellati et al., 2015; Smith et al., 2016). In contrast, the goal of this study was to conduct a systematic, in-depth, and fine-grained comparative analysis of the attributes of fibroblasts obtained from sporadic late-onset PD patients, with those from healthy age-matched control subjects. More specifically, we studied the cells from several viewpoints, including their growth dynamics and morphological characteristics, as well as mitochondrial function, redox homeostasis, and autophagy, which are core mechanisms known to be affected in PD pathogenesis. From a broader viewpoint, the identification of such cellular and molecular changes in PD fibroblasts will have significant implications toward developing diagnostic and predictive models of the disease.

## Methods

### Human subject-related information for fibroblast collection

Primary skin fibroblasts were initially generated from sun-protected skin biopsies (upper inner arm) of sporadic late-onset PD subjects with no significant family history of PD. For control comparisons, fibroblasts were acquired from similar skin biopsies of age-matched, apparently healthy individuals (age-matched controls, AMCs). The research was specifically approved by the University of Arizona Institutional Review Board, and written informed consent was obtained from all subjects before their participation. Also, in contrast to other known genetic causes of PD which result in early onset of the disease (such as PINK1, PARKIN, SNCA), since variations in the Leucine-Rich Repeat Kinase 2 **(**LRRK2) gene are known to be involved also in late-onset PD (which is the population focused upon in this study), we specifically assessed all samples for the presence of common LRRK2 mutations (Monfrini and Di Fonzo, 2017). Sequencing results indicated the absence of mutations in LRRK2 genes (Y1699C, R1441C, G2019S, and I2020T) in the collected patient fibroblasts. PCR was conducted and Sanger sequencing was carried out at the University of Arizona Genomics Core on an Applied Biosystems 3730 DNA Analyzer (Applied Biosystems, Foster City CA). Fibroblasts from PD subjects carrying a heterozygous G2019S LRRK2 mutation (obtained as described above) and homozygous G2019S LRRK2 mutation (from the NINDS Human Cell and Data repository; https://stemcells.nindsgenetics.org/?line=ND32973) were also included in some experiments for comparison. A summary of patient data related to the PD and AMC fibroblast lines used in the study is provided in Table 1 and in the Supplementary Material.

**Table 1:** Clinical information on study subjects.

	**AMC**	**PD**
Age (yrs)	64.2 ± 3.9	65 ± 5.05
N (M/F)	5 (2/3)	4 (2/2)
Time since diagnosis (yrs)	NA	7.83 ± 3.34
UPDRS (III) score	NA	8.1 ± 6.45
Daily L-Dopa (mg)	NA	456 ± 180

*Values are expressed as mean ± SD. All PD patients were taking levodopa with adjunctive dopamine agonist therapy. AMC subjects were not related to the PD subjects. One AMC subject cohabited with a PD subject*.

### Fibroblast culture

The fibroblasts were grown in highly standardized conditions using Dulbecco's modified eagle's medium (DMEM) (Thermo Fisher Scientific, Waltham MA) supplemented with 10% Fetal Bovine Serum (Atlanta Biologicals, Flowery Branch GA), 1X Non-Essential Amino Acids (Thermo Fisher Scientific) and 0.02% Primocin (InvivoGen, San Diego CA), in 5% CO_2_ at 37°C. Given that the number of passages can affect cell phenotype and responses, for all experiments in the study, passage numbers used were kept consistent within groups to avoid cell replication related biases. All experiments utilized cells from passage 4 to 14, and cells were used at ~75% confluence. The fibroblast lines were grown in parallel and assessed in at least triplicate for all experiments.

### Growth analysis

Cells were assessed when they reached ~75% confluence in culture. Two variables were measured during each passage: (1) The duration in days for the cells to reach 75% confluence; and (2) total viable cell count (using 0.4% Trypan blue staining) at 75% confluence. Population doubling was estimated using the formula Doubling time (DT) = T ln2/ln(Xe/Xb), where T is the incubation time, Xb is the cell number at the beginning of the incubation time, and Xe is the cell number at the end of the incubation time. Images were taken using a Zeiss inverted microscope with phase capability (details in Microscopy section).

### Phalloidin staining

Fibroblasts were plated at 40,000 cells/well, in 24-well plates, on poly-d-lysine (0.1 mg/ml) coated glass coverslips and subsequently fixed using 4% paraformaldehyde (PFA, Electron Microscopy Services, Hatfield PA) for 20 min at room temperature (RT). After washing with 1X Phosphate-buffered saline or PBS (Thermo Fisher Scientific, Waltham MA), the cells were treated with 0.1% Triton-X-100 (Sigma-Aldrich, St Louis MO) for 5 min. Then the cells were stained with Alexa Flour 488 Phalloidin (0.16 uM for 20 min, Thermo Fisher Scientific) and treated with 4′, 6-diamidino-2-phenylindole, dihydrochloride (DAPI, Thermo Fisher Scientific) for nuclear counterstaining.

### Morphological analysis

Fibroblasts plated at 40,000 cells/well, on poly-d-lysine coated glass coverslips placed in 24-well plates, were analyzed. Images of Phalloidin stained cells were obtained from 10 random fields/sample before processing in CellProfiler software (Broad Institute, MIT, Cambridge MA). An analysis pipeline was created to identify nuclei and cell outlines with reference to DAPI and Phalloidin staining. Images were subsequently processed to quantify area, perimeter, maximum and minimum ferret diameters, eccentricity, and form factor. The density of the fibroblast cultures was analyzed similarly via CellProfiler using images from 5 random fields/sample to generate “Percent Object Neighbors” and “Number of Adjacent Cells” data.

### UVA irradiation

UVA exposure was performed using a KW large area light source solar simulator (model 91293, Oriel Corporation, Stratford CT), equipped with a 1,000 W Xenon arc lamp power supply (model 68920) and a VIS-IR bandpass blocking filter plus UVB and C blocking filter (output 320–400 nm plus residual 650–800 nm, for UVA) (Lamore et al., 2010; Lamore and Wondrak, 2012, 2013). The output was quantified using a dosimeter (International Light Inc., Newburyport MA, model IL1700), with a SED033 detector for UVA (range 315–390 nm, peak 365 nm), at a distance of 365 mm from the source. Using a UVB/C blocking filter, the dose at 365 nm from the source is 5.39 μJ cm^−2^ sec^−1^ UVA radiation with a residual UVB dose of 3.16 μJ cm^−2^ sec^−1^. A total of 18 min exposure time per day for 4 consecutive days was used to treat the AMC and PD fibroblasts. This treatment regimen is equivalent to 5.82 J/cm^2^ UVA per day for a total of 4 days (23.76 J/cm^2^ total UVA dose).

### MTT assay

Fibroblasts were plated into 96-well plates at 10,000 cells/well (6 replicates per line), and the MTT [3-(4,5-dimethylthiazol-2-yl)-2,5-diphenyltetrazolium bromide] (Invitrogen, Carlsbad CA) assay performed. Briefly, cells were washed with PBS and placed in phenol-free culture medium. They were then treated with 12 mM MTT at 37°C for 2 h. Crystal formation was initiated by removing the MTT solution from each well and adding Dimethyl sulfoxide (DMSO; Sigma-Aldrich, St. Louis MO) after which cultures were incubated at 37°C for 10 min. The solution was vigorously mixed to solubilize crystals, and absorbance read on a standard plate reader (Synergy 2 plate reader, BioTek, Winooski VT) at 540 nm.

### Reactive oxygen species measurements

ROS was analyzed by flow cytometry using 2′,7′-dichlorodihydrofluorescein diacetate (DCFH-DA; Sigma Aldrich, St Louis MO) using previously established protocols (Lamore et al., 2010; Lamore and Wondrak, 2012). One hour after the last UVA irradiation, DCFH-DA (5 μg/mL final concentration) was added to the culture medium and cells were incubated for 1 h at 37°C and 5% CO_2_. Subsequently, the cells were harvested via enzymatic trypsinization using TrypLE Express (Invitrogen, Carlsbad CA), washed with PBS, and immediately analyzed by flow cytometry (BD FACScanto II at 488 nm excitation, 530 nm emission and CellQuest software, BD Biosciences, San Jose CA). Similarly, to measure the production of mitochondrial superoxide, cells were trypsinized and incubated in 5 μM MitoSOX Red (Invitrogen, Carlsbad CA) for 10 min. Next, the cells were washed in PBS and analyzed by flow cytometry (530 nm excitation, 593 nm emission). For both DCF and Mitosox, to avoid direct photo oxidation, cells were loaded with the indicator dye under light exclusion.

### Rhodamine 123 assay

Cells were plated on poly-d lysine (0.1 mg/ml) treated glass coverslips placed in 24-well plates at a density of 40,000 cells/well. Then the cells were treated with VectaCell Rhodamine 123 (Vector laboratories, Burlingame CA) following manufacturer's instructions. Briefly, after washing with PBS, cells were incubated with Rhodamine 123 labeling solution (diluted 1:100 in PBS) for 30 min at 37°C. Next, the labeling solution was removed, cells rinsed in PBS, and imaged immediately via confocal microscopy. Five random fields per coverslip were assessed and cells with fragmented mitochondria counted in triplicate experiments.

### Mitochondrial stress test

Cells were plated in a 96-well Seahorse XF microplate (Agilent Technologies, Santa Clara CA). Each cell line was optimized at a seeding density of 20,000 cells/well, and each consisted of a minimum of 4 wells per experimental run. Cells were incubated at 5% CO_2_ for 24 h prior to starting the experiment. Seahorse XF base medium enriched with 8 mM glucose, 5 mM l-glutamine, and 1 mM sodium pyruvate was warmed up to 37°C with an adjusted pH of 7.35 ± 0.05. All wells were washed with Seahorse medium three times while carefully making sure that the adherent cells weren't detached from the bottom of the wells. The microplate was warmed up for another 60 min in a CO_2_-free incubator before mitochondrial stress test was initiated. Successive administration of 1.0 μM oligomycin, 1.0 μM FCCP, and a combination of 1.0 μM rotenone and 1.0 μM antimycin A were mechanically done in the Seahorse XF Flux Analyzer. Respiratory control ratio (RCR), proton leak (PL, also known as state 4_O_ respiration) and coupling efficiency (CE) were calculated from the obtained oxygen consumption rates during the mitochondrial stress test.

### Cellular autofluorescence quantification

As described previously, 1 h after UVA irradiation, cells were harvested by trypsinization, washed and resuspended in PBS. They were immediately analyzed by flow cytometry (excitation 488 nm, emission 530 nm) (Lamore et al., 2010; Lamore and Wondrak, 2012).

### Western blotting

For isolating protein, fibroblasts were trypsinized, washed once in PBS, and resuspended in RIPA buffer (Sigma-Aldrich, St. Louis MO) containing Protease Inhibitor Cocktail (Sigma-Aldrich). After 1 h incubation in RIPA on ice, cells were sonicated and centrifuged at 4°C for 30 min at 15,000 × *g*. The supernatant containing the soluble protein was removed, quantified by the Lowry method, and stored at −20°C. To measure LC3 turnover, cells were incubated with 20 mM NH_4_Cl (Sigma-Aldrich) and 300 μM leupeptin (Sigma-Aldrich) for 4 h in regular medium with serum or serum-free conditions before protein samples were collected.

Protein samples from Control and PD fibroblasts were run on a 12% acrylamide gel and transferred to a PVDF membrane. After 1 h of incubation with blocking solution [0.1 M tris buffered saline (TBS) with 1% bovine serum albumin (BSA, Thermo Fisher Scientific, Waltham MA) and 5% dry milk], primary and secondary antibodies were applied. Specifically, membranes were incubated overnight in primary antibodies targeting LC3 (1:400; Cell Signaling Technology, Danvers MA) and p62 (1:500; Cell Signaling Technology) diluted in blocking solution with 0.1% Tween-20 (Sigma-Aldrich, St Louis MO). The next day, after washing in 0.1 M TBS with 0.1% Tween-20, membranes were incubated in appropriate secondary antibodies. Specifically, IRDye 680Rd (Red) or IRDye 800CW (Green) secondary antibodies at 1:10,000 (Li-Cor Biosciences, Lincoln NE) were used. All membranes were re-probed for β-Actin (1:500; Santa Cruz Biotechnology, Dallas TX) as a loading control. Proteins bands were detected using a Li-Cor Odyssey Imager and quantified using Image Studio 2.0 software. The quantitative data obtained was normalized to β-actin.

### mCherry-GFP-LC3II flux assay

Fibroblasts were grown on poly-D-lysine (0.1 mg/ml) coated glass coverslips placed in 24-well plates at a density of 20,000 cells/well for 24 h. Then, the cells were infected with Ad-mCherry-GFP-LC3 at multiplicity of infection (MOI) of 10. After 24 h post infection, the cells were washed with medium, kept in culture for another 48 h, and finally fixed in 4% PFA for confocal imaging. The number of red and yellow puncta/cell and percent area/cell covered by puncta in each cell line was enumerated using a 40x lens in seven random fields in triplicate experiments.

### Electron microscopy

Fibroblasts were plated at 1 × 10^6^ cells in a 10 mm petri dish. The cells were subsequently fixed in-situ with 2.5% glutaraldehyde in 0.1 M piperazine-N,N′-bis (2-ethanesulfonic acid) or PIPES buffer for 30 min at room temperature, and washed with 0.1 M PIPES/Glycine buffer for 10 min and PIPES for 5 min. Subsequently, the cells were post-fixed with 1% osmium tetroxide in PIPES for 30 min, washed in distilled/deionized water (DIW), scraped off well bottoms, and transferred to 2 ml microfuge tubes in which they were pelleted. Cell pellets were further washed with DIW, re-pelleted, stained with 2% uranyl acetate for 20 min and washed again in DIW. Pellets were dehydrated through an ethyl alcohol series and acetonitrile and infiltrated with 50/50 acetonitrile/ Embed 812 resin overnight. Following further infiltration with 100% resin (3 × 60 min) pellets were allowed to polymerize in the microfuge tubes overnight at 60°C. Sections of 70 nm sections were cut on a Leica Ultracut UCT ultramicrotome (Leica Biosystems, Buffalo Grove IL) onto uncoated 150 mesh copper grids and counter-stained with lead citrate. Sections were viewed in an FEI Tecnai Biotwin (FEI Company, Hillsboro OR) electron microscope operated at 100 kv. Images were collected via a 4 MP XR41 AMT side-mount camera. Morphometric measurements were conducted in digital images using Image J software (NIH). Number of autophagic vacuoles per cell profile (16–18 cell profiles) in triplicate experiments was counted.

### Microscopy

A Zeiss AxioImager A1 (Zeiss, Jena Germany) inverted microscope with phase capability, with an AxioCam MRc camera and associated AxioVision software, was used to qualitatively analyze the fibroblasts in culture. Fluorescence analysis was performed using a Zeiss M2 Imager microscope connected to an AxioCam MRm digital camera or Leica DMI6000 inverted fluorescence microscope equipped with Suite-Advanced Fluorescence 3.0 Leica Application (Leica Microsystems, Wetzlar Germany). A Leica SP5-II confocal microscope (Leica Microsystems) was used for the mitochondrial Rhodamine 123 and LC3II-mcherry-GFP assays. Z sectioning was performed at 1–2 μm intervals in order to verify the co-localization of markers. Image extraction and analysis was conducted via the Leica LAS software.

### Statistical analysis

All statistical analyses were performed using GraphPad Prism 6.0 software (GraphPad Software Inc., La Jolla CA). For normally distributed data, for comparing two groups, analysis was conducted via unpaired *t*-tests with Welch's correction. For non-normally distributed data, medians were calculated and non-parametric testing was conducting using a Mann Whitney *U*-test. For comparisons between three or more groups, analysis of variance (ANOVA) followed by Bonferroni's *post-hoc* test for multiple comparisons between groups was conducted. All data are presented as mean ± SEM, except in Figure **4J** which depicts median ± interquartile range. A *p* ≤ 0.05 was considered as significant in all cases.

## Results

### PD skin fibroblasts show distinct alterations in growth rate and spatial arrangement in culture

**Figure 1:**
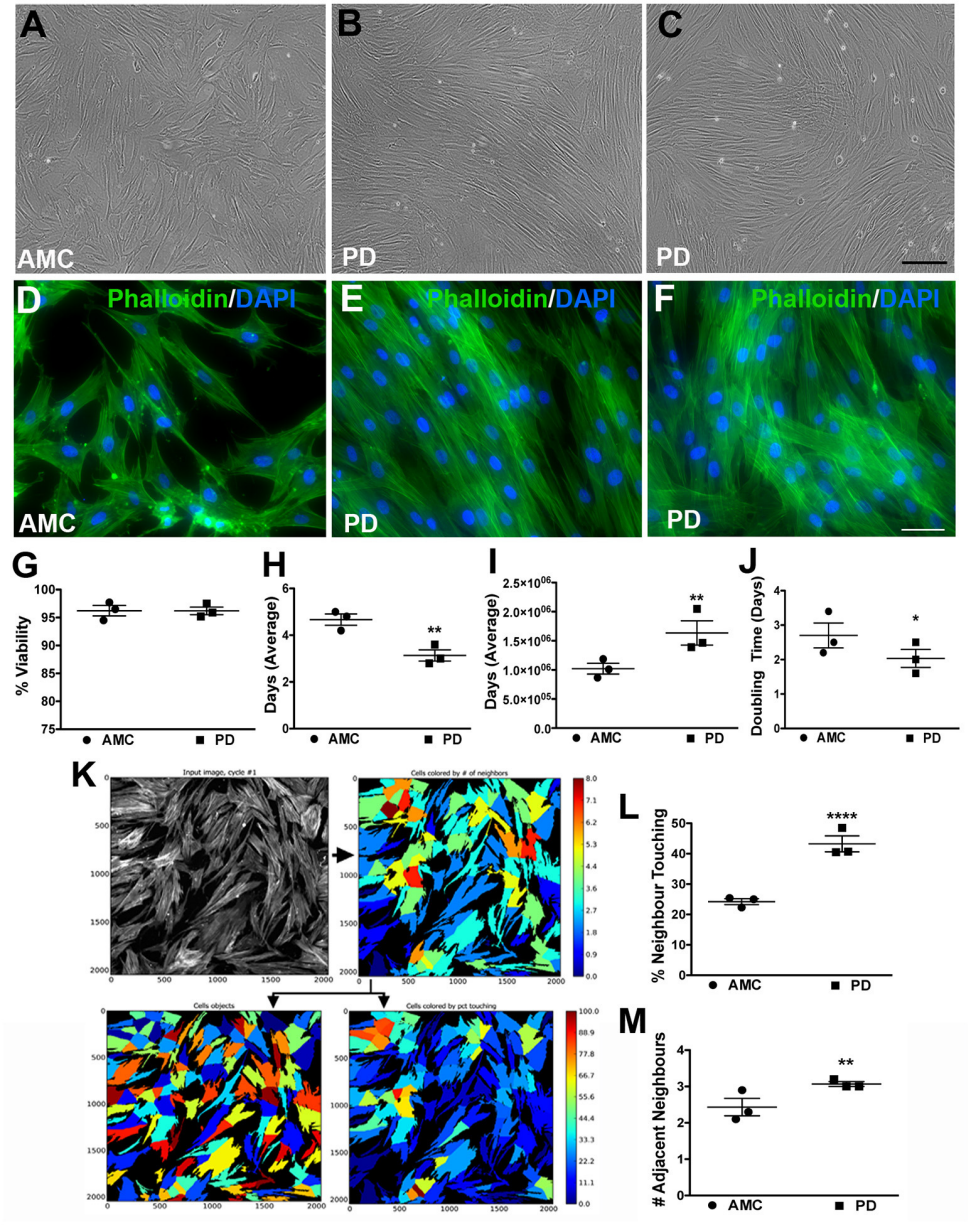
Growth and morphology differences between AMC and PD fibroblasts. PD and AMC fibroblasts exhibited distinct growth patterns in culture **(A–F)**. No differences in cell viability, assessed via a trypan blue assay, were noted between PD and AMC cultures **(G)**. However, the number of days needed to reach 75% confluence **(H)** was lower in the PD cultures. Also correlatively, a higher total cell count at the 75% confluence stage **(I)** and a higher population doubling level **(J)** was noted in the PD cultures. The density of the cultures was also quantified via CellProfiler software. **(K)** Shows how the software outlines each “cell object” based on fluorescence staining to finally measure cellular density. Comparative analysis of density between AMC and PD in terms “percentage of Neighbor Touching” and “number of Adjacent Neighbors” is shown in **(L,M)**. Scale Bars: **(A–C)** = 100 μm, **(D–F)** = 50 μm. ^*^p < 0.05, ^**^p < 0.01, ^****^p < 0.0001; Mean ± SEM, Unpaired *t*-tests with Welch's correction, *n* = 3 independent PD and AMC lines.

First, the growth characteristics of fibroblasts obtained from PD and AMC individuals were studied. It was observed that while AMC cultures exhibited features typical of mature fibroblasts, PD cultures appeared distinctly different (Figures 1A–C show phase contrast images; D-F show fluorescent images of Phalloidin/DAPI stained cells). More specifically, while AMC cells were larger, more evenly distributed, and displayed a ramified (several processes) structure, PD cells were noted to be smaller, more spindle shaped, and grouped together in a “stream-like” fashion along their longitudinal axis. Additionally, PD cultures showed higher cell densities compares to AMC cultures. These specific differences in growth and morphology between the PD and AMC cells were consistently observed across several passages in culture. Furthermore, we also compared these cell lines to PD cells with a G2019S LRRK2 homozygous (LRRK2+/+) mutation (positive control). It was observed that the LRRK2+/+ cells grew unevenly in concentrated groups in culture (Supplementary Figures 1A,D). On the other hand, cells with heterozygous G2019S (LRRK2+/−) mutation appeared qualitatively similar to sporadic PD cells (Supplementary Figures 1B,E).

To further investigate these observations, we first examined the viability of the fibroblasts using a Trypan blue assay at a stage right before passage when they had reached ~75% confluence. Our results indicate that cell viability did not differ significantly between PD and AMC fibroblast lines (Figure 1G; *p* > 0.05, Unpaired *t-*test). However, when we quantified the number of days taken to reach 75% confluence, this measure was noted to be customary (4–5 days) of growing fibroblasts in the AMC cells, but was significantly lower (2–3 days) in the PD cultures (Figure 1H; *p* < 0.01, Unpaired *t-*test). Moreover, when the total number of cells in the culture flasks was enumerated at 75% confluency, it was found that there were significantly higher numbers of cells in the PD flasks (Figure 1I; *p* < 0.01, Unpaired *t-*test). In fact, although all flasks were seeded initially with 350,000 cells during passage, the PD cells multiplied to an average of ~1.7 × 10^6^ cells/flask, compared to AMC cells which reached only 1 × 10^6^, at the 75% confluence stage (Figure 1I). Additional analysis indicated that the population doubling time (DT) of AMC fibroblasts was significantly greater at 3.02 ± 0.33 compared to a shorter 2.1 ± 0.3 in PD cells (Figure 1J; *p* < 0.05, Unpaired *t-*test). Comparatively, the DT of LRRK2+/+ cells was significantly higher (*p* < 0.05) than sporadic PD and LRRK2+/− cells (Supplementary Figure 1C, Unpaired *t-*test).

In addition, we also analyzed the density and spatial arrangement of the cells at the 75% confluence stage. Mainly, using the image analysis software CellProfiler, on Phalloidin/DAPI stained cells, we quantified the number of adjacent neighbors that each cell in culture had (Figure 1K). We also quantified the number of neighboring cells, which were in contact, or overlapping, with each other. These data showed that cells in PD flasks had about 45% of neighbors touching compared to AMC flasks where only about 20% of cells had neighbors touching (Figure 1L, *p* < 0.0001, Unpaired *t-*test). Similarly, we found that PD fibroblasts had a higher number of adjacent neighbors compared to AMC fibroblasts (Figure 1M; *p* < 0.01; Unpaired *t*-test). Overall, these data determined that the sporadic PD cultures were denser and contained more closely affiliated cells than AMC cultures. The LRRK2+/+ cultures in contrast had significantly (*p* < 0.01. Unpaired *t-*test) lower percentage of neighboring cells in contact with each other compared to sporadic PD and LRRK2+/− cells (Supplementary Figure 1F).

### PD-patient derived skin fibroblasts are morphologically different than those from healthy controls

**Figure 2:**
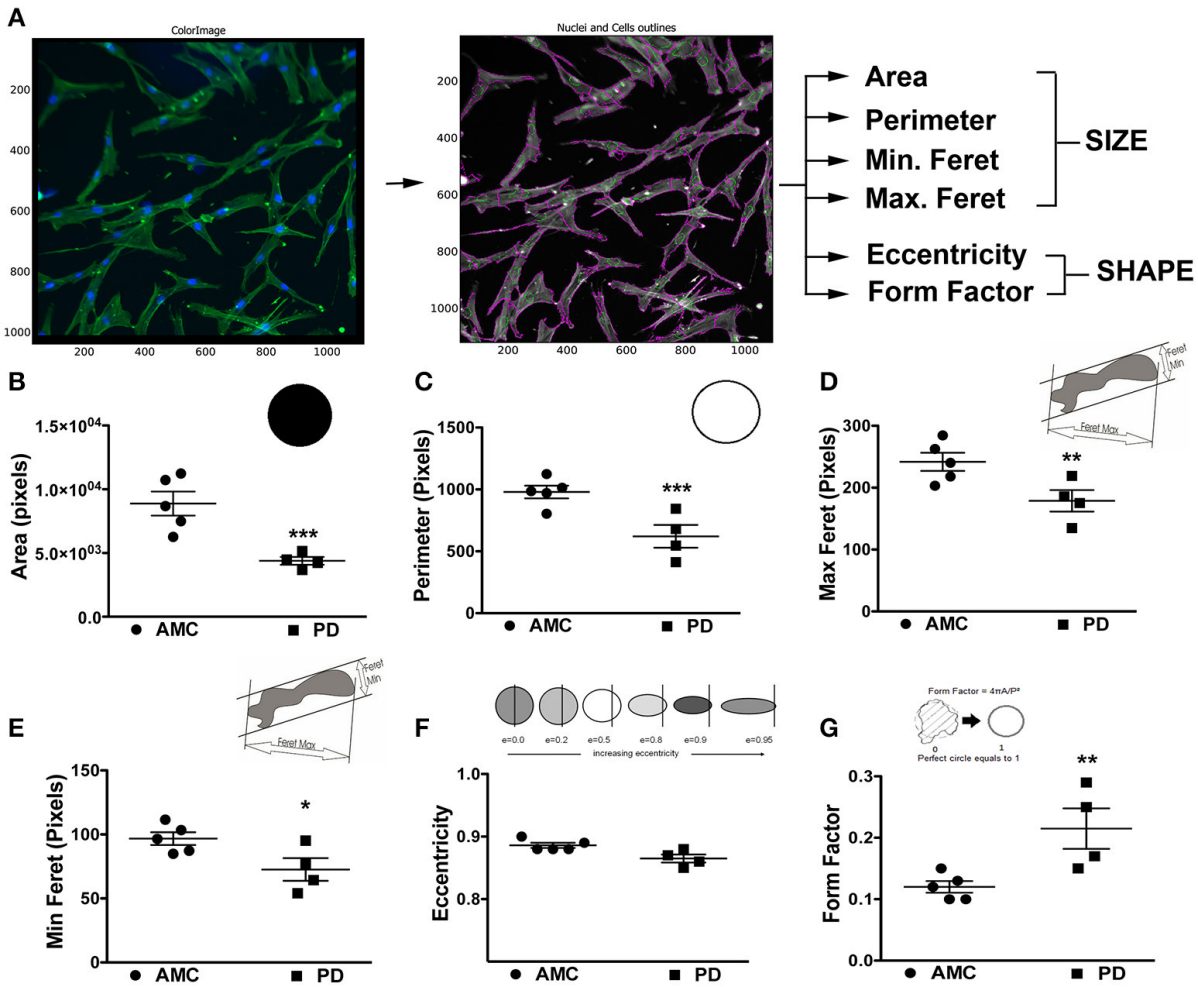
Cellular Size and Shape analysis of PD and AMC fibroblasts **(A)** Sample CellProfiler image showing outlines of “cell objects” according to fluorescence Phalloidin and Dapi stains and the list of parameters used to quantify size and shape. Quantification of cellular area **(B)**, perimeter **(C)**, maximum ferret (**D**, measuring the predicted length of the object across planes), minimum ferret (**E**, measuring the predicted width of each object across planes), eccentricity (**F**, arbitrary value for roundness), and form factor (**G**, arbitrary value on how defined the outline of an object is) in PD and AMC cultures. ^*^p < 0.05, ^**^p < 0.01, ^***^p < 0.001; Mean ± SEM, Unpaired *t*-tests with Welch's correction, *n* = 4–5 independent PD and AMC lines.

The morphology of the patient-derived fibroblasts was also examined. Specifically, the fibroblasts were stained with Phalloidin and DAPI to allow clear demarcations of cell size and shape, after which they were analyzed via the CellProfiler software (Figure 2A). Cell size, area, perimeter, and maximum and minimum feret diameters (major and minor axis of the cell) were calculated. On average, PD cells had significantly lower area (Figure 2B; *p* < 0.001, Unpaired *t-*test), perimeter (Figure 2C; *p* < 0.001, Unpaired *t-*test), and feret diameters (Figure 2D: *p* < 0.05, Unpaired *t-*test) (Figure 2E: *p* < 0.05, Unpaired *t-*test), indicating that they were smaller compared to control cells. With regards to shape, the eccentricity (a measure of cell elongation) and form factor (a measure of roundness) of the fibroblasts were measured. It was noted that although both PD and control cells were both elongated in shape (Figure 2F), PD cells were significantly more defined (that is less ramified) than AMC fibroblasts (Figure 2G; *p* < 0.01, Unpaired *t-*test). Interestingly, it was determined that the LRRK2+/+ fibroblasts were significantly (*p* < 0.001, Unpaired *t-*test) smaller (lower area and perimeter), and less defined (reduced form factor, *p* < 0.001, Unpaired *t-*test) compared to sporadic PD fibroblasts. They were also significantly (*p* < 0.05, Unpaired *t-*test) less elongated compared to both PD and AMC cells (Supplementary Figures 1G–J). These data altogether supported our qualitative observations (Figures 1A–F, Supplementary Figures 1A,B,D,E), demonstrating that the morphology of PD fibroblasts was indeed different than AMC fibroblasts.

### PD skin fibroblasts show an increased susceptibility to oxidative stress after UVA exposure

**Figure 3:**
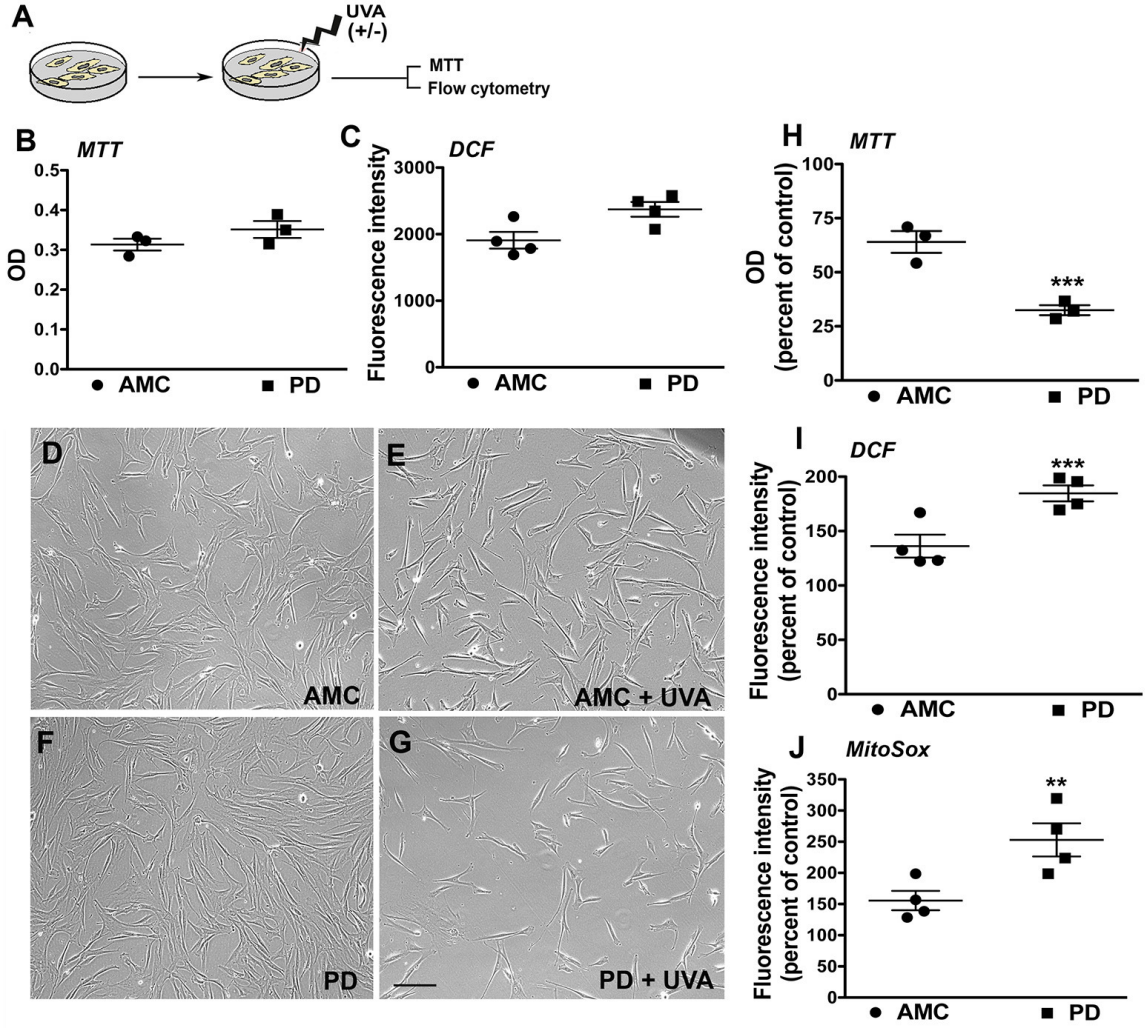
Reduced viability and increased oxidative stress in PD cultures after UVA. **(A)** Schematic of the experimental design. Baseline MTT and DCF analysis is depicted in **(B,C)**. Phase contrast images of PD and AMC cells before and after UVA treatment is shown in **(D–G)**. Comparative viability of PD and AMC cells after UVA via an MTT assay is depicted in **(H)**. Quantification of DCF-DA **(I)** and Mitosox **(J)** fluorescence post-UVA, shows greater ROS production in PD lines Scale Bars: **(B–E)** = 200 μm. ^*^p < 0.05, ^**^p < 0.01, ^***^p < 001, **(B,C)** unpaired *t*-tests with Welch's correction, **(H–J)** One way ANOVA with Bonferroni's *post-hoc* test, Mean ± SEM, *n* = 3–4 independent PD and AMC lines.

The patient fibroblasts were also exposed to ultraviolet (UV) irradiation, an environmentally relevant stressor. Specifically, the cells were treated with UVA, which is the more deeply penetrant type of solar UV radiation and constitutes most of the UV energy to which human skin is exposed (>95%, 320–400 nm) (Figure 3A; Lamore et al., 2010). UVA is also a known inducer of oxidative stress and skin photo aging (Lamore et al., 2010; Lamore and Wondrak, 2012, 2013). In particular, we applied a subchronic UVA regimen that delivered a physiologically appropriate dose, without overt cell loss in AMC cells. The viability and production of ROS in the AMC and PD fibroblasts were subsequently measured. Firstly, it was found that under baseline conditions, although there were no differences in viability (MTT cytotoxicity assay, Figure 3B), the differences in total cellular ROS levels (indicated by DCFH-DA fluorescence which represents peroxide species levels, Figure 3C) were notable although not statistically significant (*p* = 0.052) between the AMC and sporadic PD cultures. In contrast, DCFH-DA fluorescence in LRRK2+/+ cultures was significantly (*p* < 0.05, Unpaired *t-*test) higher than AMC or LRRK2 +/− cultures (Supplementary Figure 2E). Upon UVA treatment, it was noted that there was some cell loss and shrinkage in AMC cultures, however a drastic reduction in cell survival was seen in the sporadic PD cells (Figures 3D–G). Further analysis via the MTT assay showed that the sporadic PD cells had indeed undergone a significantly greater decline in viability (~77%), in comparison with control cells (~26%) (Figure 3H, *p* < 0.001, One-way ANOVA). Moreover, flow cytometric analysis of oxidative stress showed greater shifts in ROS fluorescence intensity peaks suggesting higher UVA induced ROS production in sporadic PD cells. More specifically, PD cells exhibited significantly higher total cellular ROS (DCFH-DA fluorescence, Figure 3I) as well as mitochondria-specific ROS (indicated by Mitosox fluorescence measurements, Figure 3J). In fact, total ROS in PD cells increased to about 181%, compared to 131% in AMC cells (Figure 3J; *p* < 0.001, One-way ANOVA). The changes in mitochondrial ROS production were even more pronounced, and showed an increase to 247% in PD cells compared to 155% in AMC cells (Figure 3J; *p* < 0.01, One-way ANOVA). Overall, these data indicated that PD cells had higher baseline ROS, and were substantially more sensitive to UVA-induced oxidative stress than AMC cells.

### Mitochondrial function is impaired in PD skin fibroblasts

**Figure 4:**
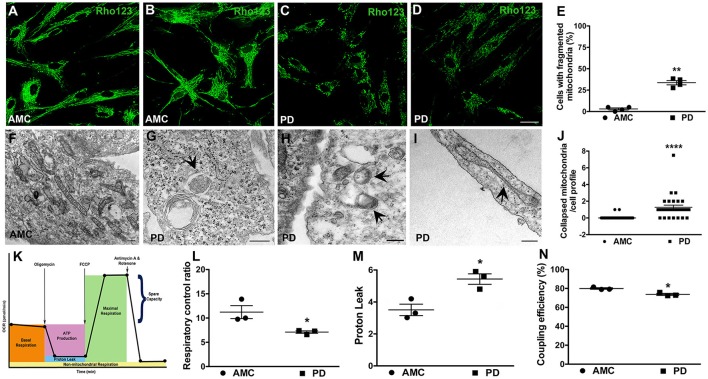
Mitochondrial dysfunction in PD fibroblasts. Rhodamine 123 stained fibroblasts showing bright and typical mitochondrial network morphology in AMC cells **(A,B)** but low intensity fragmented mitochondria in PD fibroblasts **(C,D)**. **(B,D)** are maximum intensity projection images from confocal z-stacks. Rhodamine 123 quantification is in **(E)**. Electron micrograph displaying normal mitochondrial morphology in AMC cells is depicted in **(F)**. PD cultures on the other hand showed abnormal features such as mitophagy **(G)**, mitochondria lacking cristae **(H)**, and collapsed mitochondria **(I)**. Quantification of collapsed mitochondria is in **(J)**. Schematic depicting the steps of the mitostress test is in **(K)**. RCR is significantly lower on average **(L)**, and PL is significantly higher, in PD cells **(M)**. Relatively lower CE is seen in PD lines on average **(N)**. Scale Bars: **(A–D)** = 40 μm; **(F–I)** = 250 nm. ^*^p < 0.05, ^**^p < 0.01, ^****^p < 0.0001; Mean ± SEM, Unpaired *t*-tests with Welch's correction **(E)** and two-way ANOVA with Tukey's post-test **(L–N)**; Median ± interquartile range, Mann Whitney *U*-test **(J)**; *n* = 3–4 independent PD and AMC lines.

Given the significantly higher mitochondrial ROS production noted in PD cells after UVA stress, we proceeded to examine the mitochondrial morphology and function of the patient fibroblasts in more detail. To examine morphology, we labeled fibroblast mitochondria with Rhodamine 123, a cationic fluorescent dye that labels respiring mitochondria. The dye distributes according to the negative membrane potential across the mitochondrial inner membrane. Loss of mitochondrial membrane potential results in reduced fluorescence intensity. In these experiments, it was observed that mitochondria in AMC fibroblasts had higher Rhodamine 123 fluorescence (Figure 4A). In addition, the AMC mitochondria showed normal morphology, with typical size and tubular network structure (Figure 4B—confocal maximum intensity projection image). On the other hand, mitochondria in sporadic PD fibroblasts exhibited lower Rhodamine 123 fluorescence and a fragmented appearance indicating impaired mitochondrial functioning (Figures 4C,D—confocal maximum intensity projection image). Quantitative analysis indicated that sporadic PD fibroblasts had significantly more cells with fragmented mitochondria than control fibroblasts (Figure 4E, *p* < 0.05, Unpaired *t-*test). Moreover, the LRRK2+/+ PD fibroblasts also showed lower Rhodamine 123 fluorescence (several cells showed virtually no Rh123 fluorescence retention in mitochondria (Supplementary Figure 2A; arrows) and significantly (*p* < 0.05, Unpaired *t-*test) higher numbers of fragmented mitochondria (Supplementary Figure 2F) compared to sporadic PD and LRRK2+/− cells.

Additional morphological examination via electron microscopy also supported a role for mitochondrial dysfunction in the PD fibroblasts. Here, it was observed that while AMC cells showed normal mitochondrial ultrastructure, with expected shape, size, and intact cristae (Figure 4F), mitochondria in PD fibroblasts exhibited several alterations. Specifically, smaller mitochondria lacking typical cristae (Figure 4H), as well as a number of mitochondria undergoing autophagy (enclosed within autophagic vesicles, Figure 4G) were seen in PD cells indicating ongoing “mitophagy” of subpar mitochondria. Also, mitochondria which had lost their tubular shape and appeared collapsed were predominantly noted in the sporadic PD cells (Figures 4I,J, *p* < 0.0001, non-parametric Mann Whitney *U*-test). The LRRK2+/+ cells also showed significantly more (*p* < 0.05, Unpaired *t-*test) collapsed mitochondria compared to sporadic PD and LRRK2+/− cells (Supplementary Figures 2C,D,G).

Given the morphological mitochondrial changes observed, we further assessed mitochondrial function in the sporadic PD fibroblasts by measuring oxidative phosphorylation (OXPHOS) activity using the Seahorse MitoStress test (Figure 4K; Zanellati et al., 2015; Ferng et al., 2017; Schipper et al., 2017). Three important parameters, namely respiratory control ratio (RCR), proton leak (PL), and coupling efficiency (CE), were assessed. Our data indicate that the PD cells, on average, had significantly lower RCR (Figure 4L; *p* < 0.05; Two-way ANOVA), which is the ratio between maximal uncoupled oxygen consumption (with the administration of FCCP) and state 4_O_ OCR (or proton leak). In concert with the RCR findings, proton leak (PL) was determined to be higher in PD fibroblasts compared to AMC cells (Figure 4M; *p* < 0.05; Two-way ANOVA). Furthermore, the PD fibroblasts were found to have a lower coupling efficiency suggesting that the increased PL may be the cause of lowered ATP reserve in PD cells (Figure 4N; *p* < 0.05; Two-way ANOVA). Taken together, these data supported the presence of a baseline mitochondrial dysfunction in PD fibroblasts.

### PD skin fibroblasts display altered baseline levels of autophagy

**Figure 5:**
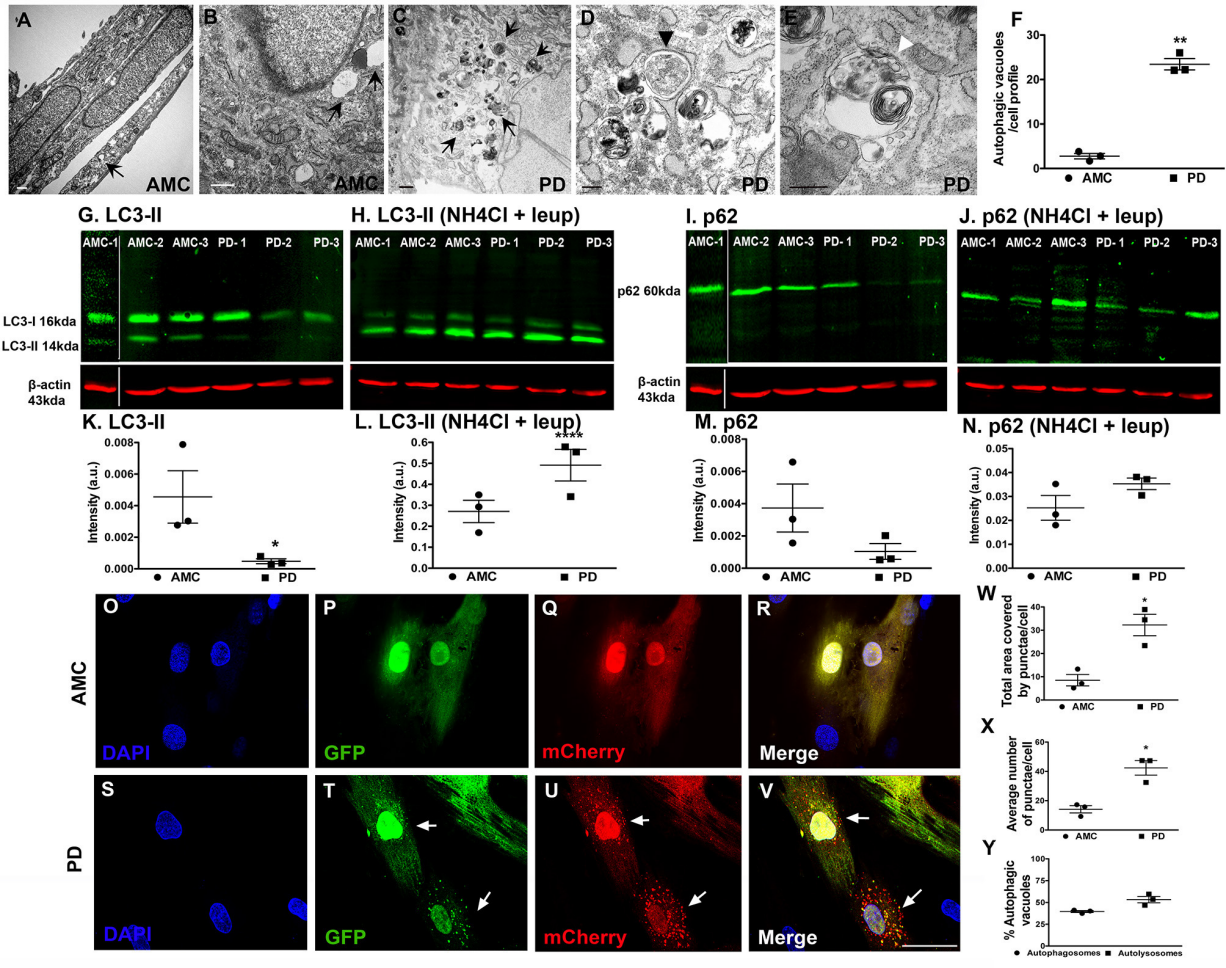
Increased autophagy at baseline in PD fibroblasts. **(A,B)** (arrows) shows electron micrographs of AMC fibroblasts with some autophagic vesicles. However, PD fibroblasts contained significantly more autophagic vesicles (**C**, arrows). A classic double-membrane autophagosome with cargo (**D**, black arrowhead) and autolysosome with degradative material (**E**, white arrowhead), as seen in a PD fibroblast. **(F)** shows EM quantification of autovesicles. Western blots showing significantly lower protein expression of autophagy marker LC3-II in PD fibroblasts **(G,K)** and p62 **(I,M)** at baseline. Normalization occurred to ß-Actin. LC3-II and p62 expression (flux) increased upon blocking of degradation via a combination of NH4Cl and Leupeptin in especially PD cell lines **(H,L,J,N)**. Fluorescence tracking of autophagic flux in Ad-mCherry-GFP-LC3 transfected AMC fibroblasts is shown in **(O–V)** with quantification in **(W–Y)**. Green puncta represent autophagosomes whereas red puncta represent autolysosomes. Scale Bars: **(A–E)** = 500 nm, **(O–V)** = 50 μm. ^*^p < 0.05, ^**^p < 0.01, ^****^p < 0.0001; Mean ± SEM, Unpaired *t-*tests with Welch's correction, *n* = 3 independent PD and AMC lines.

Next, we examined the fibroblasts in more detail via electron microscopy for evidence of autophagy, specifically macroautophagy (Schneider and Cuervo, 2014; Menzies et al., 2015). It was seen that while AMC cells showed the presence of some autophagic vesicles (Figures 5A,B; black arrows), sporadic PD fibroblasts exhibited a significantly increased collection of autophagic structures in their cytoplasm (Figure 5C, black arrows). We noted a striking accumulation of autophagic vesicles (Figure 5F, *p* < 0.01, Unpaired *t-*test), with both typical double membrane bound autophagosomes (Figure 5D; black arrowhead), as well as autolysosomes (Figure 5E; white arrowhead), in sporadic PD cells. The LRRK2+/+ cells showed the greatest changes and displayed significantly higher numbers of autophagic vesicles than AMC, sporadic PD and LRRK+/− fibroblasts (Supplementary Figures 3A–C).

To further investigate the electron microscopic findings, we examined the macroautophagy pathway more closely looking the expression of two standard autophagy markers, LC3 and p62, in the AMC and sporadic PD fibroblasts using western blotting. LC3 is involved in autophagosome formation/maturation and p62 is involved in “guiding” ubiquitinated cargo for degradation. These experiments showed that LC3 levels were significantly lower in the PD fibroblast lines as compared to AMC lines (Figures 5G,K). To understand whether the decrease in LC3II was due to the reduced production or increased degradation of LC3II, the cells were treated with a combination of ammonium chloride and leupeptin (lysosomal inhibitors) to measure autophagic flux. Upon this treatment, an increased accumulation of LC3II was noted in PD cells (Figures 5H,L) indicating higher LC3II degradation/turnover. When levels of p62 were analyzed, PD cell lines showed lower p62 expression compared to AMC lines, further supporting the notion that indeed autophagic degradation was greater in the PD cells (Figures 5I,M). This was additionally confirmed when increased p62 was noted in PD cells (Figures 5J,N) upon exposure to ammonium chloride and leupeptin. All in all, these data supported the presence of higher basal autophagy in PD fibroblasts.

We also further monitored autophagy flux in the fibroblasts through direct fluorescence microscopy by applying a viral mCherry-GFP-LC3 tandem construct. Upon transfection of this construct, it was noted that very few autophagosomes (yellow puncta) and autophagolysosomes (red puncta) were present in AMC cells (Figures 5O–R). However, substantially more autophagosomes, and autophagolysosomes, were noted in PD cells suggesting again a greater autophagic flux (Figures 5S–V). Quantitative data showed that the total area covered by puncta, as well as average number of puncta, were greater in PD cells compared to AMC cells (Figure 5W, *p* < 0.05, Unpaired *t-*test) (Figure 5X, *p* < 0.05, Unpaired *t-*test). The percentage of autophagosomes and autolysosomes were found to be comparable in the PD cells, thus indicating greater flux (Figure 5Y). In summary, these data indicated an upregulation of baseline autophagic activity in the PD fibroblasts.

### UVA promotes autophagic dysfunction in the PD skin fibroblasts

**Figure 6:**
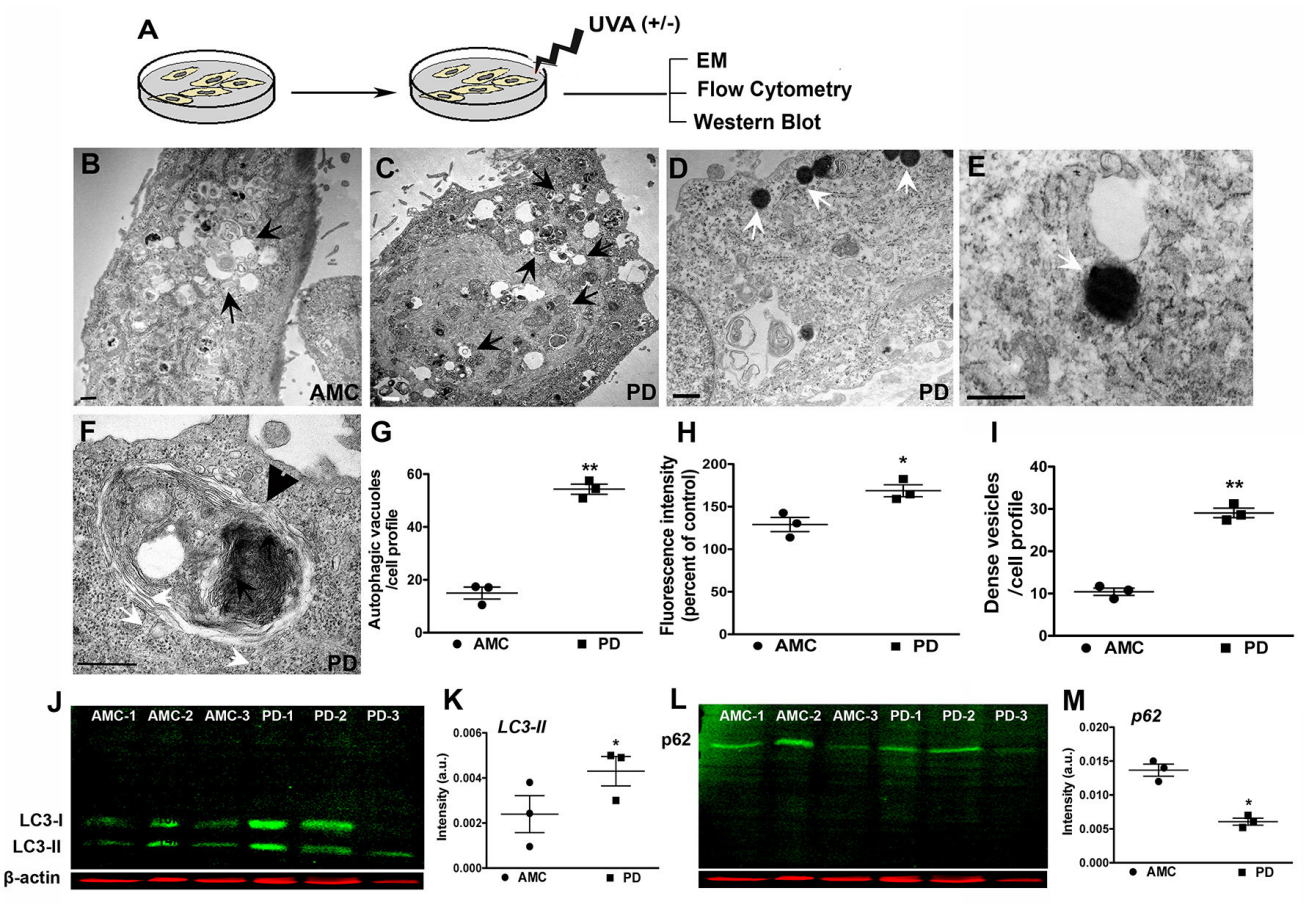
Increased autophagy in PD fibroblasts after UVA stress. **(A)** Schematic of the experimental design. Electron micrograph of a UVA-treated AMC cell showing accumulation of autophagic vesicles is in (**B**, arrows). Widespread accumulation of autophagic vesicles and lipofuscin structures is seen in PD cells (**C**, arrows). Lipofuscin-like dense structures are seen in PD cells (**D**, white arrows, and high magnification view in **E**). These are quantified in **(I)**. A high magnification image of a typical large autophagolysosome in a PD cell (**F**, black arrowhead). EM Quantification is in **(G)**. Flow cytometric analysis showed higher autofluorescence in PD lines after UVA, corresponding to presence of lipofuscin structures **(H)**. Western blotting indicated higher LC3-II expression in the PD lines **(J,K)**, and lower p62 expression, suggesting increased autophagic degradation **(L,M)**. Scale Bars: **(B–F)** = 500 nm. ^*^p < 0.05, ^**^p < 0.01, **(G)** one-way ANOVA with Bonferroni's *post-hoc* test, **(I)** Mean ± SEM, unpaired *t-*tests with Welch's correction, *n* = 3 independent PD and AMC lines.

Next, we assessed autophagy after exposure to subchronic UVA treatment which has previously been shown to cause autophagic-lysosomal blockade in human dermal fibroblasts (Lamore et al., 2010; Lamore and Wondrak, 2012, 2013; Figure 6A). Electron microscopic analysis showed an increased presence of autophagic vesicles in the AMC fibroblasts upon UVA exposure (Figure 6B; black arrows). However, an even greater autophagic response was noted in PD cells, where a widespread and exaggerated collection of autophagic structures was seen (Figures 6C–F; arrows). Quantitative analysis confirmed the significantly increased presence of autophagic structures in PD cells (Figure 6G; *p* < 0.01, Unpaired *t-*test). Furthermore, in addition to typical autophagosomes and autophagolysosomes (Figures 6C,F; black arrows, black arrowhead showing high magnification of an autophagolysosome) a marked increase in very dense structures, potentially compatible with lysosomes containing undegraded residues, was seen broadly in PD cells (Figures 6D,E,I; white arrows; Bianchi et al., 2013; Biswal et al., 2016). Given the autofluorescent nature of lipofuscin, flow cytometry was used to analyze autofluorescence levels in the fibroblasts. These results determined a significant increase in autofluorescence in PD cells after UVA exposure, compared to AMC fibroblasts, thus supporting the electron microscopic observations (Figure 6H; *p* < 0.001, Unpaired *t*-test). Moreover, we also investigated LC3 and p62 expression after UVA treatment via western blotting. Here an increase in LC3II steady state (Figures 6J,K), and a decrease in p62 (Figures 6L,M), was seen in PD cells confirming an upregulation of autophagic processing in response to UVA irradiation.

## Discussion

The development of clinically relevant biomarkers poses an enormous challenge in PD. PD's unknown multifactorial etiology, protracted course across aging, heterogeneity, and fluctuating clinical phenotype, make it a difficult disorder to diagnose. As a result, idiopathic PD usually remains unrecognized until late in its progression when most of the brain's affected neurons have already been lost, and classical motor symptoms such as tremor and rigidity have already appeared (Dexter and Jenner, 2013; Poewe et al., 2017). Moreover, even at this late stage, it is known that the sensitivity of PD clinical diagnosis in symptomatic patients is only about 90% or lower (Hughes et al., 2002; Michell et al., 2004). Hence there is a critical need for biomarkers that can reliably identify early stages of PD, and stratify this heterogeneous disease into clinical sub-types, so that new treatments can effectively be applied. We propose that the complex and varied nature of this disorder demands not one, but a “constellation” of biomarkers with diagnostic and prognostic capacities. In this context, in the current study, we report several distinct cellular and molecular alterations in skin fibroblasts obtained from idiopathic PD patients, which mimic core mechanisms characteristically seen in degenerating PD neurons. We propose that the further investigation of these fundamental fibroblast alterations will support the establishment of a novel platform to develop preclinical diagnosis and progression biomarkers for PD.

Firstly, we found that the PD fibroblasts exhibited distinct growth and morphology characteristics, not shared by cells originating from apparently healthy individuals. Specifically, our data determine that PD cells divided more rapidly than control cells. The sporadic PD cells were also smaller, more defined, and grew in tightly packed “school of fish” patterns, thus allowing individual cells to occupy lesser surface area and providing more room for density-dependent doubling. Additionally, the PD cells were more spindle-shaped, and exhibited reduced contact inhibition (were surrounded by more neighboring cells) that is characteristic of actively dividing fibroblasts. These data are supported by Cartelli et al. (2012) who also reported similar morphological changes attributed to reduced microtubular mass and changes in microtubular stability-related signaling pathways in PD fibroblasts (Cartelli et al., 2012). Interestingly, the LRRK2+/+ fibroblasts showed features distinct from sporadic PD (and AMC) cells, in that they grew more slowly and in groups or “bunches,” were larger, less defined and less elongated. These data are also supported by results in Cartelli et al. (2012) study where a similar morphology of LRRK2 cells was observed. One factor contributing to these growth and morphology changes in PD fibroblasts could be the increased ROS levels (discussed in the next paragraph) noted in the PD cells. In fact, ROS is known to affect cellular homeostatic process such as cell proliferation which in turn can also cause cytoskeletal changes (Schieber and Chandel, 2014). Cytoskeletal destabilization, affecting both the microtubule and actin structure, has been implicated as a major player that paves the way for neurodegeneration in PD (McMurray, 2000; Arduíno et al., 2012; Pellegrini et al., 2017).

Secondly, PD fibroblasts showed a greater propensity to accumulate ROS/oxidative stress, and exhibited mitochondrial and autophagic dysfunction. These three interrelated processes, namely oxidative stress, mitochondrial compromise, and autophagic dysregulation, are known to constitute core pathogenic mechanisms in PD (Sherer et al., 2002; Malkus et al., 2009; Schapira and Jenner, 2011). In terms of oxidative stress, it was observed that the PD cells displayed higher baseline ROS levels, and greater ROS accumulation upon exposure to UVA, a natural age-related environmental stressor for fibroblasts. In fact, both DCF-DA and Mitosox fluorescence were noted as significantly higher in the sporadic PD skin fibroblasts compared to AMC cells. This ROS amplification probably contributed to the significantly reduced viability of the PD cells after UVA exposure. Furthermore, the pronounced increase seen specifically in Mitosox fluorescence supported a role for mitochondria-based ROS species in this process.

In this context, it is known that mitochondria are key sources of reactive species (Sherer et al., 2002; Betarbet et al., 2006; Cookson, 2012). Mitochondrial electron transport chain disturbances can allow electrons to be transferred and reduce molecular oxygen to form superoxide and/or hydrogen peroxide. Most importantly, mitochondria play critical roles in regulating cellular energy needs and viability. Therefore, a functional impairment of mitochondria can have a severe impact on cellular homeostasis (McCoy and Cookson, 2012). Our results showed that PD fibroblasts have lower respiratory control ratio (RCR) relative to AMC cells, which indicates the reduced efficiency of mitochondria to oxidize substrates and produce ATP. Also, the higher proton leak (PL) in PD fibroblasts provides a logical explanation for the reduced RCR since a leaky membrane that leads to proton loss in the OXPHOS circuitry can cause mitochondrial inefficiency. Moreover, the reduced coupling efficiency (CE) in PD cells, relative to the AMC cells, further supports the correlation demonstrated by RCR and PL. Coupling efficiency is a measure of the fraction of protons used for mitochondrial ATP production proportional to protons leaking through the mitochondrial inner membrane, and hence serves as a supporting parameter for RCR and PL levels. Finally, we also observed structural mitochondrial alterations consistent with the functional findings. Specifically, the fragmented mitochondrial morphology and reduced fluorescence observed in PD cells (including LRRK2+/+ cells), via rhodamine 123 staining, suggested problems with mitochondrial fission/fusion and in maintaining optimal mitochondrial membrane potential. The ultrastructural findings showing collapsed mitochondria in PD cells also point toward compromised mitochondrial fusion/fission. Furthermore, the ultrastructural data, showing the loss of mitochondrial cristae and mitophagy, also support less than optimal mitochondrial respiration, mitochondrial dysfunction, and reduced mitochondrial viability, in the PD cells.

A large body of literature reveals that mitochondrial dysfunction is an important feature of degenerating neurons in PD (Sherer et al., 2002; Betarbet et al., 2006; Malkus et al., 2009; Cookson, 2012). Our observations, both biochemical and morphological, are interesting in that we see “PD-like” mitochondrial dysfunction in a peripheral non-neuronal cell such as a fibroblast. This potentially implies that there may be a global mitochondrial defect occurring in PD, which is also expressed outside the nervous system. Damaged mitochondria and oxidative stress can lead to cytoskeletal destabilization, which although needs to be confirmed, maybe a contributor to the growth and morphological changes observed in the PD cells in this study (Esteves et al., 2014).

Impaired autophagy is known as an important process underlying several neurodegenerative diseases, including PD, as evidenced by the collection of toxic, aggregate-prone, intracytosolic proteins in afflicted cells (Cuervo et al., 2010; Menzies et al., 2015). Previous evidence also suggests that the auto-activated compensatory mechanism of degradation could explain the increased number of autophagic vacuoles in the brains of PD patients (Anglade et al., 1997; Sanchez-Perez et al., 2012). In support, a similar observation has been found in PD cellular and animal models in which there were more autophagic vacuoles (Zhu et al., 2007; Choi et al., 2010; Dehay et al., 2010; Chew et al., 2011). Nevertheless, it is also known that this compensatory increase in autophagy may eventually be difficult to sustain, resulting in the build-up of toxic aggregates and neuronal death (Tanik et al., 2013). Our results reflecting a widespread appearance of autophagic vacuoles, changes in autophagy proteins, and mitophagy indicate that the PD fibroblasts may rely on such a compensatory mechanism of degradation. As expected, upon a UVA challenge, the PD fibroblasts show a further exaggerated autophagic response, which is however unable to counteract the increased accumulation of cellular materials and the loss of cell viability. Interestingly, the observation of this important PD mechanism in a peripheral cell, such as a fibroblast, may be suggestive of a more systemic cellular and molecular impairment in PD that extends beyond neurological compartments such as in the skin.

Finally, although our data as such provide the first comprehensive and detailed characterization of sporadic PD dermal fibroblasts, from several relevant cellular and molecular points of view, a limitation of the study is the relatively small sample size. Moreover, it would be relevant to investigate whether these cellular and molecular changes noted in the PD fibroblasts mimic alterations in induced dopaminergic (iDA) neurons derived from the patient fibroblasts. Nevertheless, this study does provide robust evidence that sporadic PD fibroblasts can exhibit distinct phenotypic changes, thus suggesting that phenotypes reported in PD afflicted neurons may not be totally cell specific. In essence, the reported data provide a strong foundation to further and more targetedly explore these findings in a larger and more varied population involving PD as well as other neurodegenerative disease subjects.

## Conclusion

In conclusion, our data suggest that patient-derived human dermal fibroblasts may represent a powerful model to study PD. There is a need in the field for accessible disease- and patient- specific model systems that capture the dynamic aging nature of PD. Specifically, an ideal model system should be reflective of disease status and progression while demonstrating the fundamental features and mechanisms associated with PD neuropathology. Our data indicate that PD skin fibroblasts satisfy some of these important requirements. They represent an easily accessible sample source, which reflects PD molecular changes seen in degenerating dopaminergic neurons. While other studies have shown changes in PD fibroblasts, our work presents the first comprehensive analysis of these cells from sporadic PD subjects at multiple levels, and identifies several specific characteristics that mark PD cells. More importantly, at a conceptual level, our data indicate that basic mechanisms active in neural cells in PD are likely expressed in other non-neuronal cells suggesting a generalized biological defect in PD. If such a general defect does exist, it will provide a robust platform for developing peripheral biomarkers of the disease in cells such as fibroblasts, which allow monitoring of disease progression in PD by correlating the clinical phenotype with ongoing cellular and molecular changes.

## Author contributions

JT: Experimental design, collection and assembly of data, data analysis and interpretation, manuscript writing; VB, KK, and MC: Collection and assembly of data, data analysis and interpretation, manuscript writing; RJ and AF: Collection and assembly of data, data analysis, manuscript review and critique; GW: Conception and design, collection and assembly of data, data analysis and interpretation, contributed reagents and equipment, manuscript writing; AA: Collection and assembly of data, data analysis, manuscript writing; DS: Experimental design, collection and assembly of data, data analysis and interpretation, manuscript review and critique; ZK: Experimental design, contributed reagents and equipment, manuscript review and critique; JS: Clinical support for collection of skin samples, manuscript review and critique; CC-L: Clinical support for patient recruitment and collection of skin samples, manuscript review and critique; SS: Experimental design, clinical support for patient recruitment and collection of skin samples, manuscript review and critique; LM: Conception and design, collection and assembly of data, data analysis and interpretation, manuscript writing and editing, final approval of manuscript, financial support.

### Conflict of interest statement

The authors declare that the research was conducted in the absence of any commercial or financial relationships that could be construed as a potential conflict of interest.
