# Supplementary figures and images for: Parkinson's Disease Skin Fibroblasts Display Signature Alterations in Growth, Redox Homeostasis, Mitochondrial Function, and Autophagy
Source: Front Neurosci. 2018 Jan 12;11:737. doi: 10.3389/fnins.2017.00737 (PMC5770791; doi:10.3389/fnins.2017.00737)

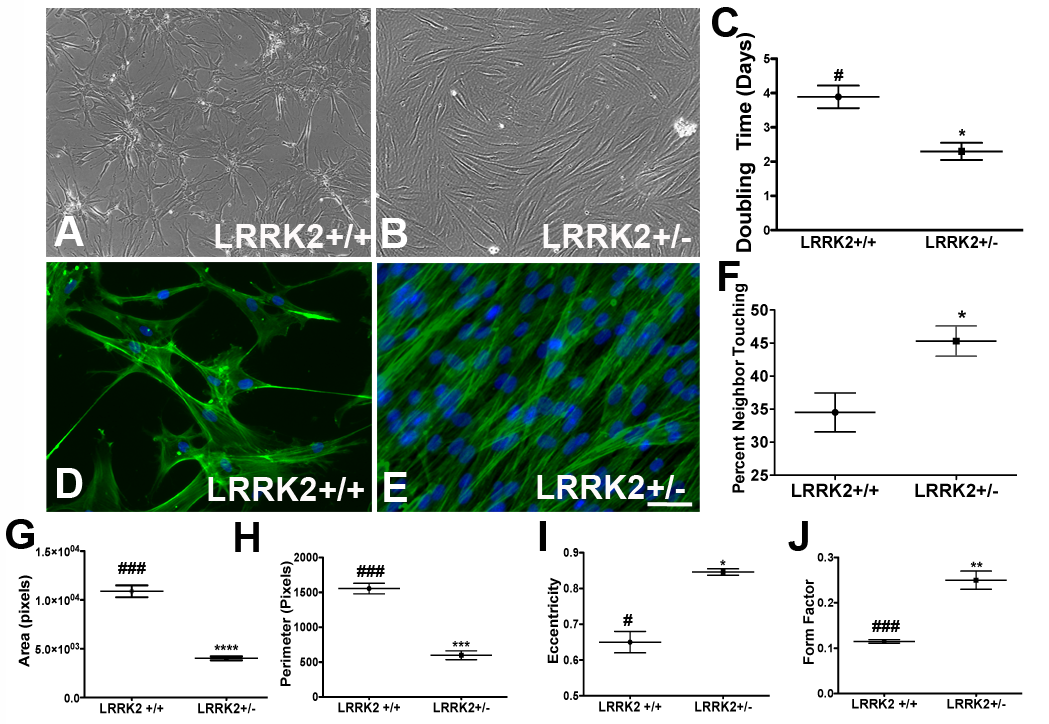

Supplement: Supplementary file 1 [file Image1.TIF]

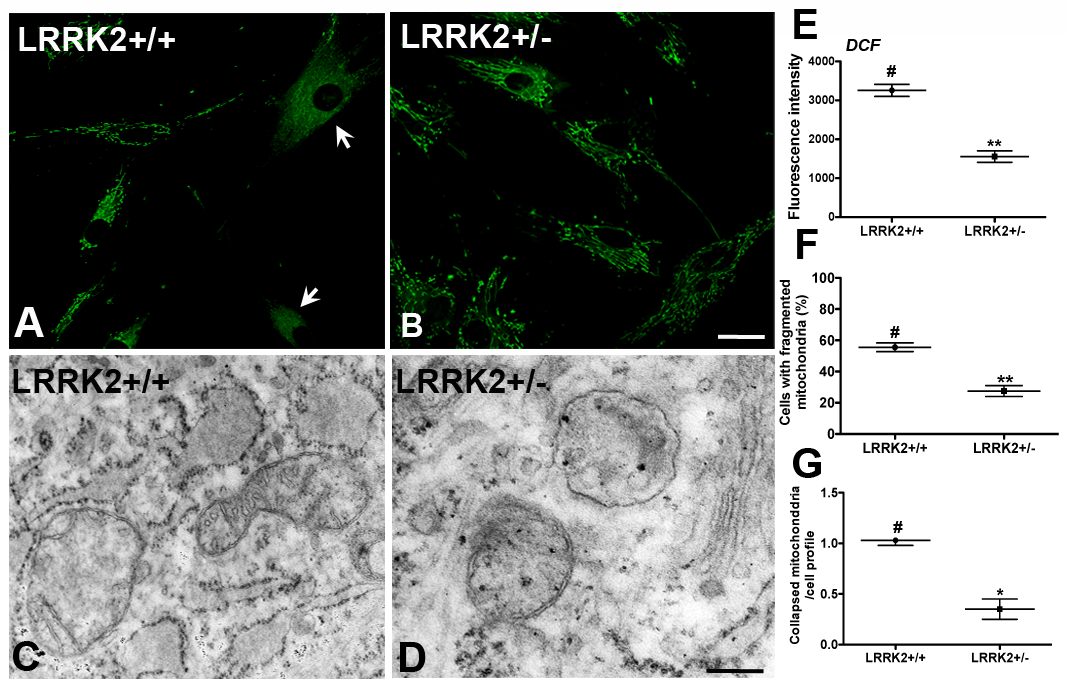

Supplement: Supplementary file 2 [file Image2.TIF]

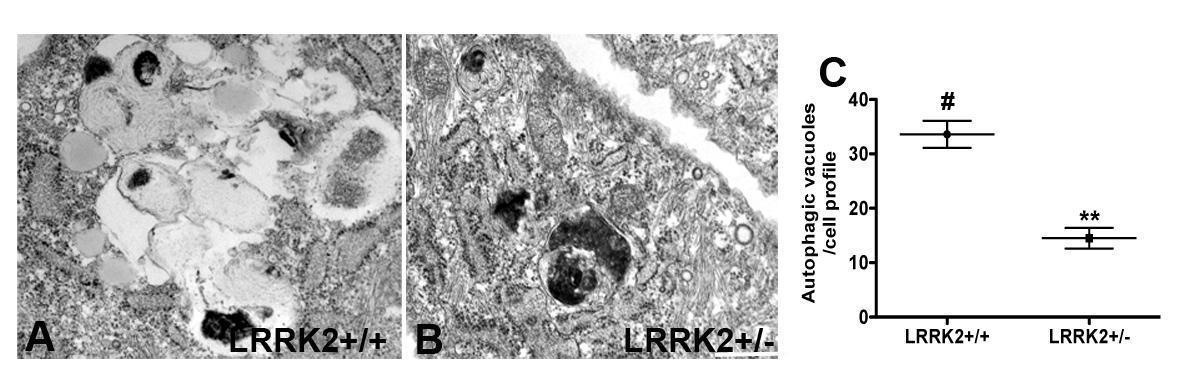

Supplement: Supplementary file 3 [file Image3.TIF]
