# Supplementary material for: Parkinson's Disease Skin Fibroblasts Display Signature Alterations in Growth, Redox Homeostasis, Mitochondrial Function, and Autophagy
Source: Front Neurosci. 2018 Jan 12;11:737. doi: 10.3389/fnins.2017.00737 (PMC5770791; doi:10.3389/fnins.2017.00737)
Supplement: Supplementary file 4 [file DataSheet1.DOCX]

**LEGENDS**

**Supplementary Figure 1: LRRK2 mutated fibroblasts show specific alterations in growth and morphology.** Qualitative representations of LRRK2+/+ and LRRK2+/- cultures in phase and after Phalloidin staining are shown in A-B, D-E. LRRK2+/+ cells showed higher doubling times and lower number of cellular neighbors touching compared to sporadic PD and LRRK2+/- cells (C, F). The LRRK2 cells were larger (G, H), less eccentric and more ramified compared to other PD cells (I, J). Scale Bars: A, B=100µm; C, D=50µm. (#p<0.05, ##p<0.01, ###p<0.0001 compared to sporadic PD; *p<0.05, **p<0.01, ****p<0.0001 compared to LRRK2+/+; Mean ± SEM, unpaired *t* tests with Welch’s correction.)

**Supplementary Figure 2: LRRK2 cells show significant changes in ROS production and mitochondrial morphology.** LRRK2 cells showed reduced Rhodamine 123 fluorescence (particularly missing in several LRRK2+/+ cells, arrows) and fragmented mitochondrial morphology (A, B, F). These LRRK2+/+ cells also had greater numbers of collapsed mitochondria (C, D, G) and increased ROS levels compared to other PD cells (E).

Scale Bars: A, B=0µm; C, D=250nm. (#p<0.05, ##p<0.01, ###p<0.0001 compared to sporadic PD; *p<0.05, **p<0.01, ****p<0.0001 compared to LRRK2+/+; Mean ± SEM, unpaired *t* tests with Welch’s correction.)

**Supplementary Figure 3: LRRK2 cells exhibit increased autophagy.** A and B depict EM level accumulations of autophagic vesicles in the LRRK2+/+ and LRRK2+/- cells. Graph in C indicates that the LRRK2 +/+ cells have a higher concentration of autophagic vesicles compared to other sporadic and LRRK2+/- PD cells. Scale Bars: A, B=500nm. (#p<0.05 compared to sporadic PD; **p<0.01 compared to LRRK2+/+; Mean ± SEM, unpaired t tests with Welch’s correction.)

**TABLE 2: Clinical information on LRRK2 subjects**

| **Table 2: Clinical Information on LRRK2 subjects** | | |
| --- | --- | --- |
|  | LRRK2 G2019S Het | LRRK2 G2019S Homo |
| Age (yrs) | 67 | 72 |
| Sex | M | M |
| Time since diagnosis (yrs) | 3 | 10 |
| UPDRS (III) score | 2 | Not available |
| Daily L-Dopa (mg) | Not available | Not available |

**Full Blots for Figures 5 and 6**

**Corresponding to last 5 lanes in Figures 5G and 5I**


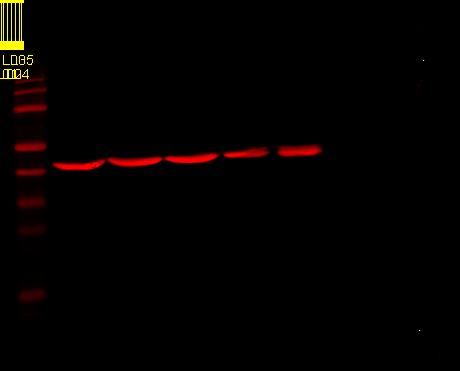

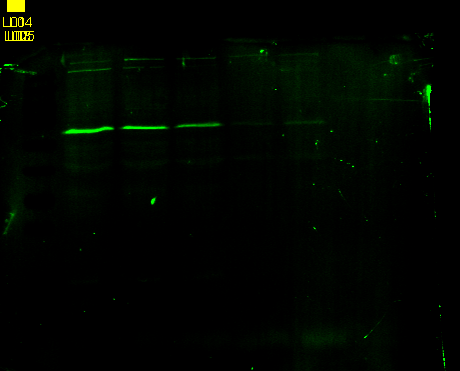

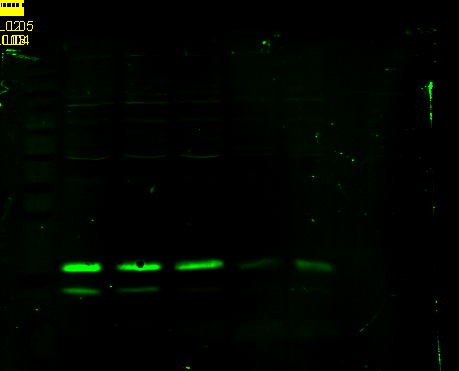
**LC3 P62 B-Actin**

**Corresponding to Figures 5H and 2J (the membrane was cut to a smaller size before probing for p62)**

**
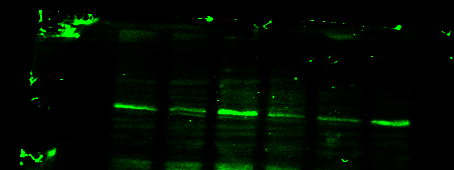

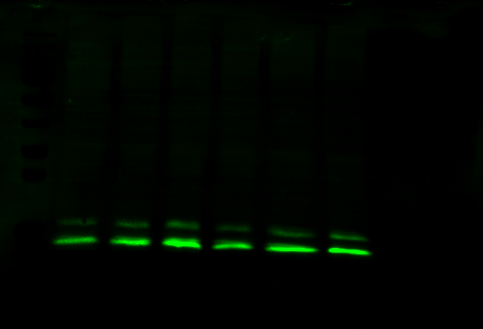

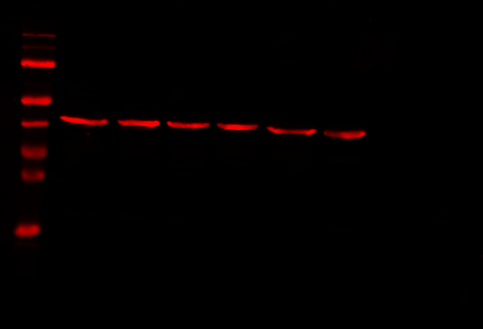
LC3 P62 B-Actin**

**Corresponding to Figure 6H and J. The last lane, after the marker, corresponds to lane 1 in figure 5G and I.**

**
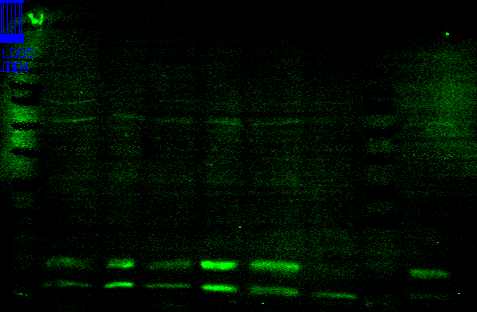

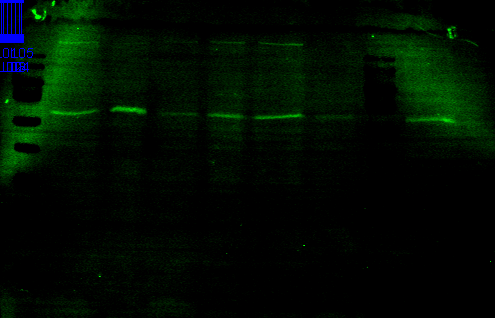
**
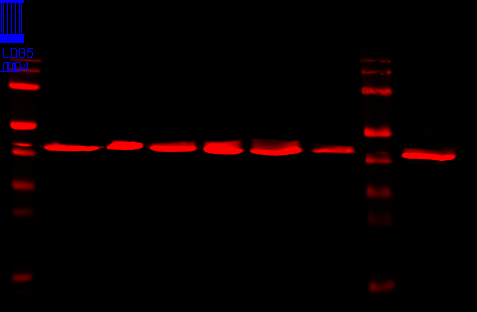
**LC3 P62 B-Actin**

**Repeat of blots in Fig 6H and J (UVA treated AMC and PD samples)**

**LC3 P62 B-Actin**


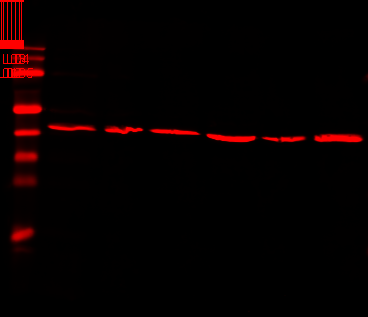

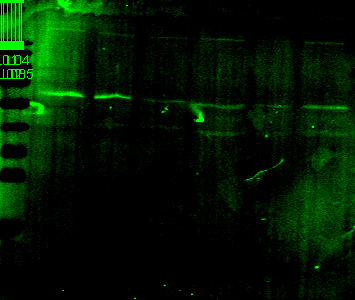

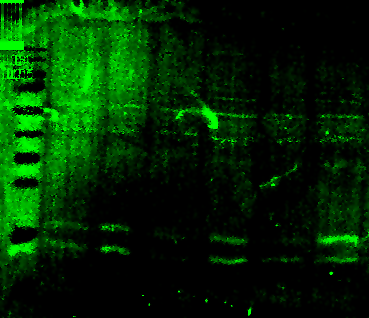
 AMC1 AMC2 AMC3 PD1 PD2 PD3 AMC1 AMC2 AMC3 PD1 PD2 PD3 AMC1 AMC2 AMC3 PD1 PD2 PD3
